# Cross-reactions between engineered xylose and galactose pathways in recombinant *Saccharomyces cerevisiae*

**DOI:** 10.1186/1754-6834-3-19

**Published:** 2010-09-01

**Authors:** Rosa Garcia Sanchez, Bärbel Hahn-Hägerdal, Marie F Gorwa-Grauslund

**Affiliations:** 1 Department of Applied Microbiology, Lund University, P.O. Box 124, SE-22100 Lund, Sweden

## Abstract

**Background:**

Overexpression of the *PGM2 *gene encoding phosphoglucomutase (Pgm2p) has been shown to improve galactose utilization both under aerobic and under anaerobic conditions. Similarly, xylose utilization has been improved by overexpression of genes encoding xylulokinase (XK), enzymes from the non-oxidative pentose phosphate pathway (non-ox PPP) and deletion of the endogenous aldose reductase *GRE3 *gene in engineered *Saccharomyces cerevisiae *strains carrying either fungal or bacterial xylose pathways. In the present study, we investigated how the combination of these traits affect xylose and galactose utilization in the presence or absence of glucose in *S. cerevisiae *strains engineered with the xylose reductase (XR)-xylitol dehydrogenase (XDH) pathway.

**Results:**

In the absence of *PGM2 *overexpression, the combined overexpression of XK, the non-ox PPP and deletion of the *GRE3 *gene significantly delayed aerobic growth on galactose, whereas no difference was observed between the control strain and the xylose-engineered strain when the *PGM2 *gene was overexpressed. Under anaerobic conditions, the overexpression of the *PGM2 *gene increased the ethanol yield and the xylose consumption rate in medium containing xylose as the only carbon source. The possibility of Pgm2p acting as a xylose isomerase (XI) could be excluded by measuring the XI activity in both strains. The additional copy of the *PGM2 *gene also resulted in a shorter fermentation time during the co-consumption of galactose and xylose. However, the effect was lost upon addition of glucose to the growth medium.

**Conclusions:**

*PGM2 *overexpression was shown to benefit xylose and galactose fermentation, alone and in combination. In contrast, galactose fermentation was impaired in the engineered xylose-utilizing strain harbouring extra copies of the non-ox PPP genes and a deletion of the *GRE3 *gene, unless *PGM2 *was overexpressed. These cross-reactions are of particular relevance for the fermentation of mixed sugars from lignocellulosic feedstock.

## Background

The use of renewable lignocellulosic biomass as raw material for the production of second-generation bioethanol is an alternative to sugar- and starch-containing feedstocks, which are also used for food and feed production. Lignocellulosic biomass can contain a significant fraction of pentose sugars such as xylose [1] that is not naturally fermented by the preferred microorganism for industrial bioethanol production, baker's yeast *Saccharomyces cerevisiae *[2]. Therefore recombinant xylose-fermenting *S. cerevisiae *strains have been generated to increase the overall ethanol yield from lignocellulosic biomass [3].

Xylose utilization by *S. cerevisiae *has been achieved either by expression of the *P. stipitis XYL1 *and *XYL2 *genes encoding the NAD(P)H-dependent xylose reductase (XR) and the NAD^+^-dependent xylitol dehydrogenase (XDH) [4-6] or by expression of genes encoding xylose isomerase (XI) [7,8]. When compared in an isogenic strain background, the XI strain displayed higher ethanol yield, whereas the XR-XDH strain had higher ethanol productivity [9]. Furthermore, the engineering of several additional steps has been shown to benefit xylose conversion to ethanol, including the overexpression of the xylulokinase (XK) gene [10,11], the overexpression of genes encoding the enzymes of the non-oxidative pentose phosphate pathway (non-ox PPP) [12,13] and the deletion of the aldose reductase gene encoded by *GRE3 *[14]. In the context of lignocellulose fermentation, these genetic modifications must be evaluated in the presence of other lignocellulose derived sugars (glucose, galactose, mannose and arabinose) and together with genetic modifications that favour the utilization of these sugars. In the case of galactose, the overexpression of the *PGM2 *gene encoding phosphoglucomutase has been shown to improve galactose utilization both under aerobic [15] and under anaerobic conditions [16].

In the current investigation, we therefore evaluated genetic modifications improving xylose and galactose utilization separately and in combination in *S. cerevisiae *strains expressing the XR-XDH pathway. The resulting strains were characterized in medium containing xylose, a mixture of either xylose and galactose or of xylose, galactose and glucose.

## Results

### Overexpression of the *PGM2 *gene in xylose-utilizing strains

To evaluate the influence of *PGM2 *overexpression on xylose utilization, the *PGM2 *gene was chromosomally integrated under the control of the truncated *HXT7 *promoter [17] in strain TMB 3320, already harbouring the *P. stipitis *XR-XDH pathway and genetic modifications that improve xylose consumption: high XK and high non-ox PPP activity as well as deletion of the *GRE3 *gene. The resulting strain was named PGM2-PPP-XYL (Table 1). The corresponding control strain lacking only the extra copy of *PGM2 *gene was also constructed and named Control-PPP-XYL (Table 1). Additionally, strains lacking the overexpression of non-ox PPP genes and the deletion of the *GRE3 *gene were constructed and named Control-XYL and PGM2-XYL (Table 1).

**Table 1:** *S. cerevisiae *strains and plasmids used in this study

*S. cerevisiae *strains and corresponding abbreviations	Strain background	Relevant genotype/phenotype	Reference
**TMB 3320**	TMB 3043	CEN.PK2-1C, Δ*gre3 his3::HIS3 PGK1p-XK1-PGK1t*, *PGK1p-TAL1-PGK1t*, *PGK1p-RKI1-PGK1t*, *PGK1p-TKL1-PGK1t, PGK1p-RPE1-PGK1t, TRP1, leu2::LEU2 ****ADH1p-XYL1-ADH1t PGKp-XYL2-PGKt ura3***	Garcia Sanchez et al., manuscript in preparation
**CEN.PK 113-11C**		*MAT***a ***his3Δ1 ura 3-52 MAL2-8c SUC2*	[42]
**TMB 3137 "Control-PPP-XYL"**	TMB 3320	*ura3::URA3 *YIplac211	This work
**TMB 3138 "PGM2-PPP-XYL"**	TMB 3320	*ura3::URA3 *YIplac211 HXT-PGM2	This work
**TMB 3141**	CEN.PK 113-11C	*his3:: HIS3 *YIpXR/XDH/XK	This work
**TMB 3139 "Control-XYL"**	TMB 3141	*his3:: HIS3 *YIpXR/XDH/XK *ura3::URA3 *YIplac211	This work
**TMB 3140 "PGM2-XYL"**	TMB 3141	*his3:: HIS3 *YIpXR/XDH/XK, *ura3::URA3 *YIplac211 HXT-PGM2	This work
**TMB 3128 "Control m"**	CEN.PK 113-11C	*his3::HIS3 *YEplacHXT *URA3*	[16]
**TMB 3129 "PGM2 m "**	CEN.PK 113-11C	*his3::HIS3 *YEplacHXT7'p-PGM2 *URA3*	[16]
**TMB 3135 " Control i"**	CEN.PK 113-11C	*his3::HIS3 ura3::URA3*	[16]
**TMB 3136 "PGM2 i"**	CEN.PK 113-11C	*his3::HIS3 ura3::URA3 *Ylplac211 HXT-PGM2	[16]
**Plasmids**			

**YIplac211**		*URA3*	[43]
**YIplac211 HXT-PGM2**		YIplac211, *HXT7'p-PGM2-PGKt URA3*	This work
**YIpXR/XDH/XK**		*ADHp-XYL1-ADHt, PGKp-XYL2-PGKt, PGKp-XK-PGKt, HIS3, β-lactamase*	(Eliasson, Christensson et al. 2000)

### Aerobic growth on xylose and galactose

The four xylose-utilizing strains were compared with respect to aerobic growth on 50 g l^-1 ^xylose to assess how overexpression of *PGM2 *affected xylose utilization. In agreement with their genetic makeup, strains overexpressing the non-ox PPP genes and carrying a deletion of the *GRE3 *gene (Control-PPP-XYL and PGM2-PPP-XYL) had a three- to four-fold higher growth rate on xylose than strains Control-XYL and PGM2-XYL (Table 2). On the other hand, *PGM2 *overexpression did not significantly affect the aerobic growth on xylose.

**Table 2:** Maximum specific growth rate (_μ_max ± standard deviation) on 50 g l^-^^1 ^galactose or 50 g l^-^^1 ^xylose under aerobic conditions and using YNB medium.

	Carbon source	
Strain	**50 g l**^**-1 **^**galactose**	**50 g l**^**-1 **^**xylose**
**Control-PPP-XYL**	0.21 ± 0.01	0.038 ± 0.014
**PGM2-PPP-XYL**	0.32 ± 0.02	0.041 ± 0.008
**Control-XYL**	0.23 ± 0.01	0.012 ± 0.002
**PGM2-XYL**	0.27 ± 0.01	0.012 ± 0.003
**Control m**	0.22 ± 0.01 (*)	ND
**PGM2 m**	0.30 ± 0.02 (*)	ND
**Control i**	0.24 ± 0.02 (*)	ND
**PGM2 i**	0.32 ± 0.01 (*)	ND

(*) Taken from [16]. ND: Not determined. For both carbon sources, the inocula were prepared in defined medium with 20 g l^-1 ^glucose.

**Figure 1:**
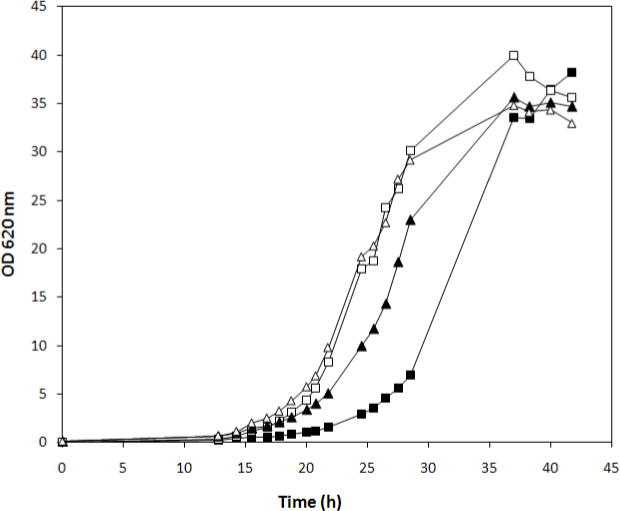
**Aerobic growth in batch culture on YNB medium with 50 g l**^**-1 **^**galactose with inocula grown in YNB medium with 20 g l**^**-1 **^**glucose**. *S. cerevisiae *strains: *Control-PPP-XYL *(filled square), *PGM2-PPP-XYL *(open square), *Control-XYL *(filled triangle), *PGM2-XYL *(open triangle).

The four xylose-utilizing strains were also compared with respect to aerobic growth on 50 g l^-1 ^galactose (Figure 1). We confirmed in both strain backgrounds that galactose utilization was increased by overexpression of the *PGM2 *gene (Figure 1; Table 2). Strains PGM2-XYL and PGM2-PPP-XYL differed slightly in their maximum specific growth rate (μ_max_) values: 0.27 ± 0.01 and 0.32 ± 0.02 h^-1 ^(Table 2), respectively. These μ_max _values are in the same range as those of the two previously described *S. cerevisiae *strains overexpressing only *PGM2*, PGM2 m and PGM i [16] (Table 1 and 2). In contrast, the absence of *PGM2 *overexpression delayed growth on galactose for both xylose-utilizing strains, significantly more for strain Control-PPP-XYL than for strain Control-XYL (Figure 1), whereas their maximum specific growth rates were similar and in the same range as previously described for strains Control i and Control m, 0.21 and 0.23 h^-1 ^respectively, [16] (Table 1 and 2). When *PGM2 *was overexpressed, the difference between the two strains disappeared and the lag phase was shortened (Figure 1).

### Anaerobic fermentation of xylose

**Figure 2:**
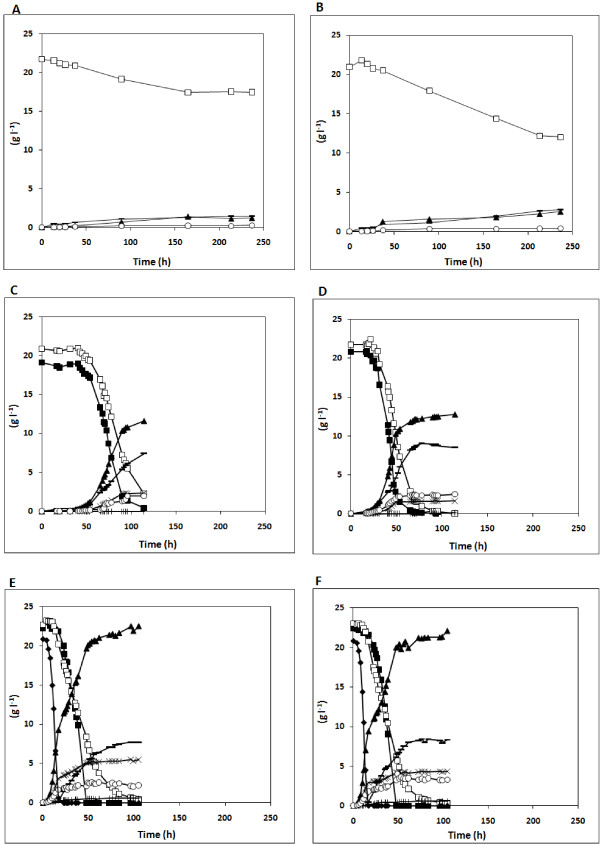
**Sugar consumption and product formation during anaerobic batch fermentation in defined medium**. Strains: *Control-PPP-XYL *(A, C, E) and *PGM2-PPP-XYL *(B, D, F) with 20 g l^-1 ^xylose (A, B), 20 g l^-1 ^xylose and 20 g l^-1 ^galactose (C, D) or 20 g l^-1 ^xylose, 20 g l^-1 ^galactose and 20 g l^-1 ^glucose (E, F). Inocula were grown in defined medium with 20 g l^-1 ^glucose. Symbols: galactose (filled square), xylose (open square), glucose (filled diamond), ethanol (filled triangle), biomass (DW) (open circle), acetate (plus symbol), glycerol (multiplication symbol), xylitol (minus symbol).

The effect of *PGM2 *overexpression on xylose utilization was also compared under anaerobic conditions with strains Control-PPP-XYL and PGM2-PPP-XYL (Figures 2A and 2B). In contrast to aerobic conditions, the μ_max _in 20 g l^-1 ^xylose medium was 1.9-fold higher (*p-value *< 0.09) for the strain overexpressing the *PGM2 *gene: 0.023 ± 0.002 h^-1 ^vs. 0.012 ± 0.004 h^-1^. This strain also consumed 112% more xylose than the control strain (Figures 2A and 2B). The increased xylose consumption resulted in a two-fold increase in biomass and a 20% increase in ethanol yield, whereas glycerol and xylitol yields decreased five- and two-fold, respectively (Table 3).

**Table 3:** Parameters analyzed during anaerobic batch fermentation on different sugar mixtures.

		Yield			
Carbon source	Strain	^**a **^**Biomass g/g substrate**	^**a **^**Glycerol g/g substrate**	^**b **^**Xylitol g/g xylose**	^**a **^**Ethanol g/g substrate**
**Xylose**	Control-PPP-XYL	0.040 ± 0.006	0.056 ± 0.017	0.29 ± 0.04	0.25 ± 0.06
	PGM2-PPP-XYL	0.076 ± 0.002	0.011 ± 0.000	0.15 ± 0.07	0.30 ± 0.06

**Galactose + Xylose**	Control-PPP-XYL	0.056 ± 0.007	0.061 ± 0.004	0.29 ± 0.05	0.31 ± 0.02
	PGM2-PPP-XYL	0.064 ± 0.004	0.042 ± 0.001	0.37 ± 0.04	0.32 ± 0.00

**Galactose + Xylose + Glucose**	Control-PPP-XYL	0.061 ± 0.002	0.138 ± 0.028	0.39 ± 0.04	0.32 ± 0.00
	PGM2-PPP-XYL	0.070 ± 0.001	0.115 ± 0.003	0.40 ± 0.03	0.32 ± 0.01

^**a**^Biomass, glycerol and ethanol yields were calculated by plotting the consumed sugar/s (g l^-1^) against the produced biomass (g biomass l^-1^), the produced glycerol (g glycerol l^-1^) or the produced ethanol (g ethanol l^-1^) respectively on the phase of constant and highest values. ^**b**^Xylitol yields were calculated by plotting the consumed xylose (g xylose l^-1^) against the produced xylitol (g xylitol l^-1^) on the phase of constant and highest values.

### Anaerobic fermentation of mixed sugars

Anaerobic fermentation of a mixture of xylose and galactose was further investigated for strains Control-PPP-XYL and PGM2-PPP-XYL (Figures 2C and 2D; Table 3). In the presence of galactose, the xylose consumption rate increased for both strains compared to when xylose was the sole carbon source (Figures 2A and 2B). Moreover, xylose and galactose were co-consumed, with galactose being the preferred sugar. When the *PGM2 *gene was overexpressed, the fermentation of both sugars proceeded faster (Figure 2D). In addition, the biomass yield increased slightly, whereas the glycerol yield decreased 1.5-fold and the xylitol yield increased 1.3-fold (Table 3).

The two strains were then investigated in anaerobic fermentation of a mixture of xylose, galactose and glucose (Figures 2E and 2F; Table 3). As expected, the repressing sugar glucose was consumed first and xylose and galactose consumption started only when the glucose concentration was below 5 g l^-1^. In contrast to the fermentation of the xylose and galactose mixture (Figures 2C and 2D), xylose started being consumed before galactose while galactose was more readily depleted.

In the presence of glucose, the fermentation time was almost identical for the two strains, regardless of the presence of an additional copy of the *PGM2 *gene. Also glycerol, xylitol and ethanol yields were similar for the two strains, whereas the biomass yield increased 1.15-fold in the *PGM2*-overexpressing strain. Glucose fermentation increased the glycerol yield two- to three-fold.

### XI activity

To rule out that the increased growth on xylose arose from a XI activity of Pgm2p, the XI activity was determined in galactose-grown cells of strains Control m and PGM2 m. The PGM activity had previously been shown to be five to six times higher in the strain carrying an extra copy of *PGM2 *integrated on the genome as compared to the control strain [16]. We were not able to detect any XI activity in cell extracts of either strain Control m or strain PGM2 m (data not shown).

## Discussion

The present study investigated the cross-reactions that may occur in *S. cerevisiae *when two independent genetic engineering strategies for the improvement of the utilization of two different carbon sources, namely, galactose and xylose, were combined. The work demonstrated that *PGM2 *overexpression improved not only galactose but also anaerobic xylose utilization in recombinant xylose-utilizing *S. cerevisiae *strains. The beneficial effect was also observed in mixtures of xylose and galactose, but it disappeared in the presence of glucose. In parallel, the improved aerobic growth on galactose as a result of *PGM2 *overexpression, which had previously been observed in different strains [15,16], was maintained in xylose-utilizing strains. In contrast, aerobic growth on galactose was significantly delayed in the engineered xylose-utilizing strain harbouring extra copies of the non-ox PPP genes and a deletion of the *GRE3 *gene, unless *PGM2 *was overexpressed.

The anaerobic growth rate on xylose increased up to 1.9 times when *PGM2 *was overexpressed. In addition, the total amount of consumed xylose increased 2.1-fold (Figure 2) and the ethanol yield was 20% higher (Table 3). *PGM2 *upregulation has been observed in several genome-wide transcription analyses of strains developed for improved xylose utilization. The *PGM2 *level was 4.8- and 3.9-fold higher on xylose and xylose-glucose media under aerobic conditions and 1.6-fold higher on glucose-xylose medium under anaerobic conditions in an anaerobically growing evolved strain as compared to the non-growing parental strain [18]. In another genome-wide transcription analysis that compared several improved xylose-growing *S. cerevisiae *strains, all structural genes from the galactose pathway were derepressed, resulting, among others, in *PGM2 *upregulation [19]. Still, there is no immediate connection between the enzyme PGM2 and xylose metabolism. We assayed cell extracts for XI activity and confirmed that PGM2 is an intramolecular transferase isomerase [20,21] rather than an intramolecular oxidoreductase isomerase such as XI [22,23].

**Figure 3:**
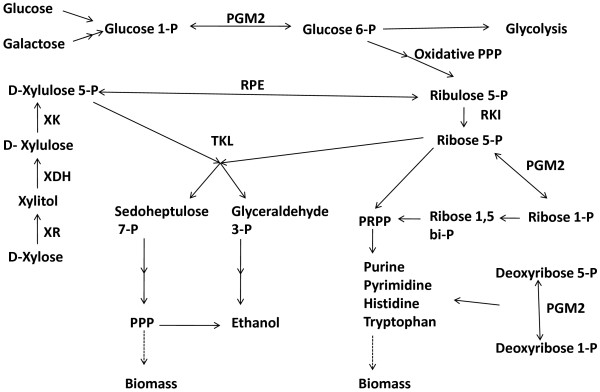
**Metabolic reactions involving the PGM2 (adapted from **[41]). Abbreviations: PPP, Pentose phosphate pathway; PRPP, phosphoribosyl pyrophosphate; RPE, ribulose 5-phosphate 3-epimerase; RKI, ribose 5-phosphate ketol-isomerase; TKL, transketolase; -P, phosphate.

Another connection between PGM2 activity and xylose metabolism could be that PGM2 catalyzes phosphotransferase reaction(s), which enhance anaerobic growth by increasing the pool of intermediary metabolites required for biosynthetic reactions. Phosphomannomutase activity has been demonstrated for *S. cerevisiae *PGM1 and PGM2 [24]. Furthermore, the human homolog to *S. cerevisiae *PGM2 has been shown to be more active as phosphopentomutase than as phosphoglucomutase [20]. It could notably use ribose 1-P, ribose 5-P, deoxyribose 1-P and deoxyribose 5-P as substrates [20], suggesting that *S. cerevisiae *PGM2 may be able to convert ribose 5-P to ribose 1-P and vice versa (Figure 3). Xylose is catabolized via ribose 5-P, an intermediate of the non-ox PPP, which via ribose 1-P is a precursor of histidine and tryptophan biosynthesis as well as of purine, pyrimidine and ultimately nucleic acid biosynthesis (Figure 3). *PGM2 *overexpression may thus increase the xylose flux and pull xylose catabolism by converting ribose 5-P to ribose 1-P and further to biomass formation (Figure 3). In fact, increased biomass production was obtained, regardless of carbon source, when the *PGM2 *gene was overexpressed (Table 3). Despite a large standard deviation, the increased biomass formation was accompanied by a reduced glycerol formation (Table 3), which may reflect a reduced requirement for synthesis of biomass intermediates [25]. Finally, *PGM2 *overexpression reduced xylitol formation in anaerobic xylose fermentation, which serves as an additional indication of an increased xylose flux [9].

Co-fermentation of xylose and galactose increased the rate of xylose utilization compared with pure xylose fermentation. Furthermore, both sugars were depleted faster when *PGM2 *was overexpressed while the ethanol yield remained unchanged. Enhanced xylose uptake in the presence of galactose has previously been reported [26]. Galactose induces the galactose catabolizing enzymes of Leloir pathway [27], including the galactose transporter Gal2p [28]. In addition to transporting galactose, Gal2p has been shown to transport xylose [29], and its expression in *S. cerevisiae *is induced 1000-fold when the yeast is exposed to galactose [27], which was found also to benefit xylose utilization. *PGM2 *overexpression increases the intracellular pool of glucose 6-P [30], which has been shown to activate glycolysis [31-33]. However, when glucose was added to the medium, the benefit of *PGM2 *overexpression for xylose and galactose co-fermentation was abolished, in agreement with the fact that the glucose 6-P pool is higher in glucose than in galactose grown cells [34]. Thus the xylose and galactose fermentation time was reduced both by glucose and by *PGM2 *overexpression, but the effect was not additive.

## Conclusions

Genetic modifications that were known to improve individually xylose and galactose utilization were combined and evaluated on various sugar combinations. *PGM2 *overexpression was shown to benefit xylose and galactose fermentation, alone or in combination. In contrast, galactose fermentation was impaired in the engineered xylose-utilizing strain harbouring extra copies of the non-oxidative PPP genes and a deletion of the *GRE3 *gene, unless *PGM2 *was overexpressed. Knowledge on these cross-reactions is of particular relevance in the context of bioethanol production from mixtures of sugars derived from lignocellulosic feedstock.

## Methods

### Strains

*Escherichia coli *DH5α and JM101 (Life Technologies, Rockville, MD, USA) were used for cloning. Plasmids and *S. cerevisiae *strains (Table 1) were stored at -80°C in 15% glycerol. Freshly streaked plates from frozen stocks were used to inoculate precultures.

### Molecular biology techniques

Standard molecular biology techniques were used for cloning [35]. Restriction enzymes, T4-DNA ligase and Shrimp Alkaline Phosphatase (SAP) used for cloning were obtained from Fermentas (Vilnius, Lithuania). Yeast genomic DNA was purified with Easy-DNA Kit (Invitrogen, Groningen, The Netherlands). *E. coli *DH5α competent cells were prepared as described before [36] and transformed using the calcium chloride method [37]. Yeast was transformed using the lithium acetate method [38]. Plasmids were extracted from bacteria either with GeneJET TM (Fermentas, Vilnius, Lithuania) or with Qiagen Mini Plasmid Purification kit (Qiagen GmbH, Hilden, Germany). The QIAquick kit was used for gel extraction of DNA fragments from agarose gel as well as for purification of amplified DNA products. Taq polymerase (Fermentas, Vilnius, Lithuania) was used for analytical PCR, while Pfu-polymerase (Fermentas, Vilnius, Lithuania) and Pwo-polymerase (Roche Diagnostics GmbH, Mannheim, Germany) were used for high-fidelity polymerase chain reactions. The Abi-Prism Big-Dye cycle sequencing kit (Applied Biosystems, Weiterstadt, Germany) was used for DNA sequencing that was performed by BM Labbet AB (Furulund, Sweden).

### Strain construction

Yeast strains carrying an extra copy of the *PGM2 *gene under a strong constitutive promoter and the corresponding control were constructed in two strain backgrounds. First, the xylose-utilizing strain TMB 3320 was transformed with *Eco*RV-linearized plasmids YIplac211 and YIplac211 HXT-PGM2 in the *URA3 *locus, to generate strain Control-PPP-XYL, TMB 3137, and strain PGM2-PPP-XYL, TMB 3138, respectively (Table 1).

In parallel, strain CEN.PK 113-11C was transformed with plasmid YIpXR/XDH/XK (Table 1) cleaved with *Pst*I in the *HIS3 *locus. Yeast transformants that were selected in defined medium lacking histidine and supplemented with uracil were able to grow in defined medium with 50 g l^-1 ^xylose. The resulting strain TMB3141 was further transformed with plasmids YIplac211 and YIplac211 HXT-PGM2 (Table 1) that were cleaved in the *URA3 *locus with *Eco*RV. The resulting strains were named Control-XYL, TMB 3139, and PGM2-XYL, TMB 3140, respectively (Table 1). Genomic DNA was extracted from strains Control-PPP-XYL, PGM2-PPP-XYL, Control-XYL and PGM2-XYL to verify integration at the correct loci using PCR.

### Aerobic cultivation

*E. coli *was grown and selected on Luria-Bertani (LB) [35] with 100 mg/L ampicillin. For yeast growth, Yeast Nitrogen Base medium (YNB; Difco Laboratories/Becton Dickinson, Sparks, MD, USA) at a concentration of 6.7 g l^-1 ^was supplemented either with 50 g l^-1 ^galactose, 50 g l^-1 ^xylose, or 20 g l^-1 ^glucose. YNB liquid medium was buffered with potassium hydrogen phthalate (10.21 g l^-1 ^phthalate, 2.1 g l^-1 ^KOH, pH 5.5) [39]. The concentration of YNB was doubled when the sugar concentration was higher than 20 g l^-1 ^to avoid nutrient limitation. Plates were supplemented with 20 g l^-1 ^glucose and 20 g l^-1 ^agar.

Precultures were grown in YNB with 20 g l^-1 ^glucose until mid- to late exponential phase overnight in 50-ml tubes with 5 ml of growth medium. The precultures were used to inoculate aerobic batch cultures at OD_620 nm _0.1-0.2 in cotton-stoppered baffled 500-ml flasks with 50 ml of growth medium and the relevant carbon source. The medium was supplemented with amino acids when using auxotrophic strains. Precultures and aerobic batch cultivation experiments were performed at 30°C and 180-200 rpm agitation (Gallenkamp INR-200, Leicester, UK) at least in duplicate.

### Enzymatic activity

XI activity was measured in crude extracts of cells grown in YNB medium with 20 g l^-1 ^galactose using the sorbitol dehydrogenase assay [8]. Cells were harvested in exponential phase, centrifuged at 5000 rpm for 5 min, washed with cold sterile water twice and permeabilized with Y-PER (Pierce, Rockford, IL, USA). The protein concentration was determined with Coomassie Protein Assay Reagent (Pierce, Rockford, IL, USA) using bovine serum albumin as standard. Reagents used to determine enzyme activity were purchased from Sigma-Aldrich (St. Louis, MO, USA). For every strain and condition, enzyme activity measurements of at least two independent biological replicates were performed.

### Anaerobic fermentation

For anaerobic fermentation and corresponding precultivation, defined mineral medium was used [40]. The medium was supplemented with 0.4 g l^-1 ^Tween 80 and 0.01 g l^-1 ^ergosterol. The carbon source was either 20 g l^-1 ^xylose, a mixture of 20 g l^-1 ^xylose and 20 g l^-1 ^galactose, or a mixture of 20 g l^-1 ^xylose, 20 g l^-1 ^galactose, and 20 g l^-1 ^glucose. When the sugar concentration exceeded 20 g l^-1^, the concentration of Tween 80 and ergosterol was doubled.

Aerobic precultivation was performed at 30°C (Gallenkamp INR-200, Leicester, UK) and 180-200 rpm. The preculture medium contained 20 g l^-1 ^glucose in phthalate buffer as described for the aerobic cultivation. A first preculture was grown until late exponential phase in 5 ml culture in 50-ml tubes and was used to inoculate a second aerobic preculture of 100 ml in 1000-ml cotton-stoppered baffled shake flasks. The second preculture was grown until late exponential phase, washed twice with sterile water after centrifugation at 5000 rpm for 10 min and used to inoculate anaerobic batch cultures at OD_620 nm _0.1-0.2. Anaerobic batch fermentation was performed in either 3 l Biostat Bio Reactors (B. Braun Biotech International, Melsungen, Germany) or Applikon Bio Reactors (Applikon, Schiedam, The Netherlands) with a working volume of 1.5 l at 30°C and 200 rpm and pH controlled at 5.5 with 3 M KOH. Anaerobic conditions were obtained by sparging nitrogen gas containing less than 5 ppm O_2 _(AGA Gas, Sundbyberg, Sweden) at a flow rate of 0.2 l min^-1 ^controlled by a gas mass flow meter (Bronkhorst, HI-TECH, Ruurlo, The Netherlands). Outlet carbon dioxide and oxygen were monitored by a Carbon Dioxide and Oxygen Monitor type 1308 (Brüel & Kjaer, Copenhagen, Denmark). Anaerobic fermentation experiments were performed at least in duplicate.

### Analysis of metabolites

Galactose, xylose, glycerol, acetic acid and ethanol were separated by high-pressure liquid chromatography (HPLC) (Waters, Milford, MA, USA, or Beckman Instruments, Fullerton, CA, USA) with an Aminex HPX-87H ion exchange column (Bio-Rad, Hercules, CA, USA) at 45°C. The mobile phase was 5 mM H_2_SO_4 _at a flow rate of 0.6 ml min^-1^.

When xylose and galactose were present in the same sample xylose, galactose, glucose and xylitol were additionally separated and quantified by one or two in series connected HPX-87P (Bio-Rad, Hercules, CA, USA) (Waters, Milford, MA, USA) ion exchange column(s) at 85°C with water as the mobile phase at a flow rate of 0.5 ml min^-1^. A refractive index detector (RID-6A; Shimadzu, Kyoto, Japan) was used for quantification.

Cell dry weight was determined at least in duplicate at different time points during the fermentation experiment by filtering 5 ml of culture through a preweighed hydrophilic polyethersulfone 0.45 μM filter (Pall Life Sciences, Ann Arbor, MI, USA). Filters were then dried in a microwave and cooled prior to weight determination.

## Abbreviations

PGM2: phosphoglucomutase isoform 2; PPP: Pentose phosphate pathway; non-ox: non-oxidative; XR: xylose reductase; XDH: xylitol dehydrogenase; XK: xylulokinase.

## Competing interests

BHH is co-owner and chairman of the board of C5 Lignotechnologies in Lund AB.

RGS and MFGG declare that they have no competing interests.

## Authors' contributions

RGS participated in the design of the study, performed all the experimental work and drafted the manuscript. BHH and MFGG participated in the design of the study and edited the manuscript. All authors read and approved the final manuscript.
